# Dietary Habits and Their Impact on Pediatric Obesity and Asthma: A Narrative Review with Emphasis on the Mediterranean Diet

**DOI:** 10.3390/children12101354

**Published:** 2025-10-09

**Authors:** Marianna Deligeorgopoulou, Sophia Tsabouri, Ekaterini Siomou, Antonios P. Vlahos, Anastasios Serbis

**Affiliations:** Department of Pediatrics, University of Ioannina, 45110 Ioannina, Greeceeksiomou@uoi.gr (E.S.);

**Keywords:** Mediterranean diet, children, obesity, asthma

## Abstract

Obesity and asthma are increasingly prevalent chronic conditions that often coexist in the pediatric population and may influence each other through shared pathophysiological mechanisms. Obesity can affect asthma expression and severity via mechanical effects on the lungs, systemic inflammation, altered adipokine levels, and metabolic dysregulation. These mechanisms contribute to a distinct asthma phenotype in children with obesity that is often less responsive to standard therapy. Nutrition plays a critical role in this context by influencing immune function, inflammation, and respiratory outcomes. Specific dietary patterns, such as the Mediterranean diet, along with nutrients including vitamin D, antioxidants, and polyunsaturated fatty acids, have been associated with the modulation of airway inflammation and asthma risk. Additionally, early-life nutritional exposures and gut microbiota composition may influence immune development and the propensity for allergic diseases. This narrative review aims to synthesize current evidence on the interplay between obesity, asthma, and nutrition in the pediatric population, highlighting potential dietary interventions and targets for improved asthma management in children with obesity.

## 1. Introduction

Over recent decades there has been a considerable increase in the prevalence of both obesity and asthma in pediatric populations in developed and developing countries [1,2,3]. As context, the Global Asthma Network (GAN) Phase I (2015–2020) reported current asthma symptoms in 9.1% of children and 11.0% of adolescents worldwide, while the NCD Risk Factor Collaboration documented a rise in global childhood obesity from 0.7% to 5.6% in girls and 0.9% to 7.8% in boys between 1975 and 2016 [4,5]. This parallel increase in prevalence has raised concerns over the interconnection between obesity and asthma. Indeed, several longitudinal epidemiological studies and meta-analyses in adults [6] and in children, refs. [7,8,9], show that obesity often precedes asthma onset and is associated with poorer control and exacerbations, while asthma has also been linked to an increased risk of subsequent obesity [10].

Several genetic, mechanical, and environmental factors may influence the bidirectional and complex interplay between obesity and asthma [11]. Firstly, it has been shown that adipose tissue accumulation in the abdomen and chest wall of obese individuals decreases tidal volumes and increases airway resistance [12], gradually aggravating the asthma phenotype [13]. Secondly, the pro-inflammatory state that characterizes obesity is considered by many to be one of the main factors linking obesity and asthma exacerbations and severity [14]. Several inflammatory mediators such as Interleukin (IL)-6, tumor necrosis factor (TNF-α), interferon (IFN-γ), and monocyte chemoattractant protein-1 (MCP-1) have been associated with obesity and asthma [14,15,16]. In addition, insulin resistance, which characterizes individuals with obesity and which accentuates a pro-inflammatory state, has been shown to be associated with poorer respiratory function in asthmatic patients independently of body mass index (BMI) [17,18]. Furthermore, the dysregulation of adipokines, such as increased leptin and resistin and decreased adiponectin, has been observationally associated with asthma pathogenesis through inflammation and metabolic dysregulation [19,20].

In addition, evidence suggests that dietary patterns in childhood and adolescence are linked to the development of both obesity and asthma [21,22,23,24]. Specifically, the term Mediterranean diet (MD) was first described by Ancel Keys in the 1960s. It is characterized by high intakes of plant-based foods (vegetables, fruits, whole grains, legumes, and nuts), olive oil as the principal fat, moderate fish and poultry, and low intakes of red/processed meats and sweets. Given its nutrient profile (unsaturated fats, dietary fiber, and polyphenols) and lower dietary inflammatory potential, the MD has been suggested by pediatric studies [25,26] as a possible preventive or therapeutic approach for children and adolescents with obesity and asthma [27].

The current review aims to synthetize current evidence on the interplay between obesity, asthma, and the ways that the MD can positively affect both diseases and mitigate their complications, highlighting, thus, its role as an important health strategy in such pediatric populations.

## 2. Methods

This narrative review was conducted by systematically searching the PubMed and Google Scholar databases. The initial search included the following keywords: “obesity,” “asthma,” “pediatrics,” “children,” “diet,” “Mediterranean diet,” and “micronutrients.” To focus on the role of specific dietary components, additional searches were performed using terms such as “Vitamin C,” “Vitamin D,” “antioxidants,” “microbiome,” “asthma,” and “obesity.” Only articles written in English and published up to December 2024 were included.

Two authors independently reviewed the abstracts and reference lists of the selected studies to ensure no relevant articles were missed, and no disagreements arose during the selection process. The inclusion criteria were studies that specifically addressed the relationship between diet, obesity, and asthma in the pediatric population, with a particular focus on the MD and its components. Both observational and interventional studies were included, with emphasis on those that explored diet’s influence on inflammation, metabolic health, and body mass index (BMI) in children and adolescents. Studies focusing mainly on adult populations were excluded, with the exception of a limited number of investigations on micronutrients and their impact on metabolic health. Furthermore, non-English publications were excluded, as well as studies restricted to populations with severe comorbidities not generalizable to the pediatric population.

Laboratory equipment, devices, or reagents were not a part of this review. The literature search was completed using PubMed (National Center for Biotechnology Information, Bethesda, MD, USA) and Google Scholar (Google LLC, Mountain View, CA, USA). References were managed with EndNote 20 (Clarivate Analytics, Philadelphia, PA, USA).

**Figure 1:**
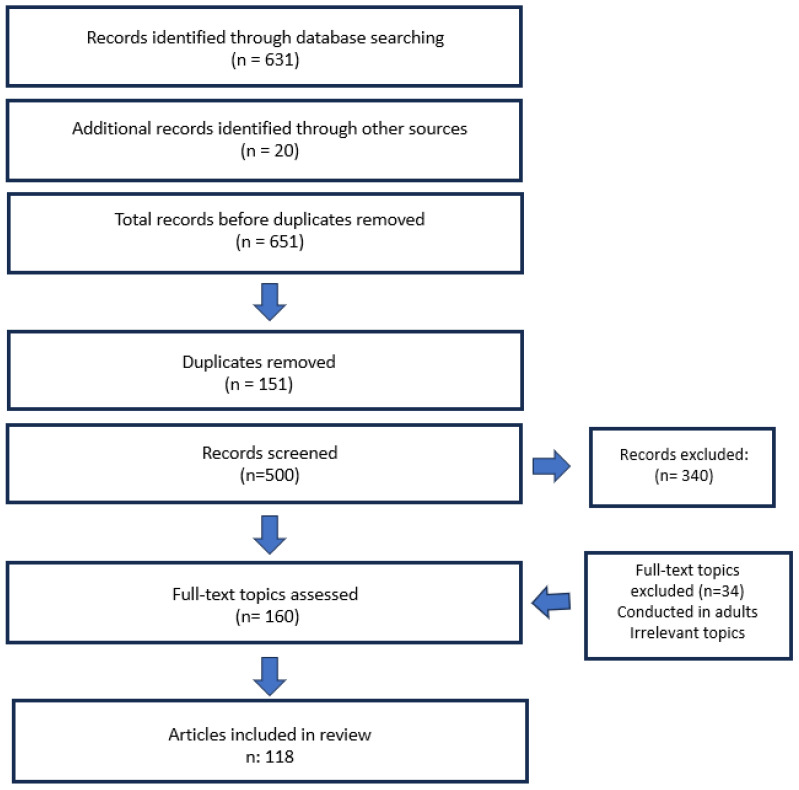
PRISMA flow diagram of the study selection process.

Figure 1 depicts the literature search and selection procedure.

## 3. Discussion

**Interconnection between obesity and asthma**

Nowadays, the dietary habits of even the populations living in Mediterranean countries are closer to the Western way of eating, characterized by an increased consumption of animal fats and a reduced consumption of fruits, vegetables, and unprocessed products. At the same time, the incidence of chronic diseases such as asthma and obesity has increased, particularly in the pediatric population [5,28]. A prospective study by Schatz et al., including 518 children aged 6–11 years and 3612 subjects older than 12 years, demonstrated that the prevalence of asthma was nearly twice as high among obese children (15.3%) compared to normal-weight participants (8%) [29].

**Figure 2:**
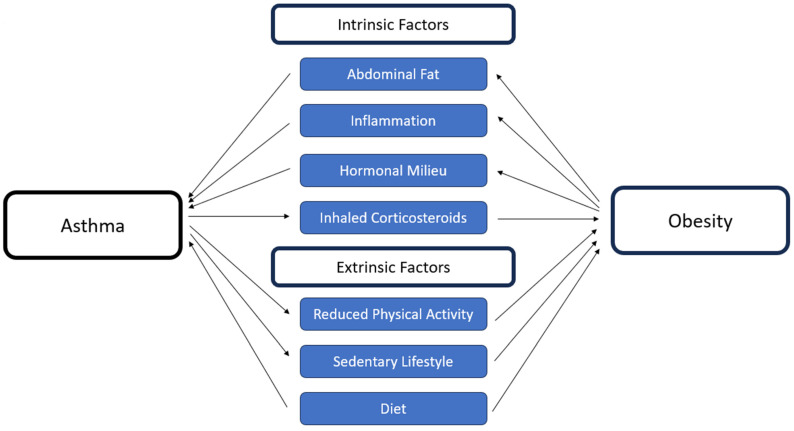
The bidirectional relationship between asthma and obesity in children, mediated through shared intrinsic and extrinsic factors. These overlapping mechanisms highlight the complexity of their interaction and the potential role of dietary interventions.

Many studies in adults and children point to a possible shared pathophysiology linking obesity and asthma, with multiple parameters investigated and discussed in more detail in the sections below (Figure 2) [30].

### 3.1. Intrinsic Factors

#### 3.1.1. Mechanical Factors

Initially, the accumulation of fat in the abdomen and chest wall is considered a mechanical cause correlating obesity and asthma. This condition leads individuals with obesity to breathe in smaller tidal volumes, adopting a shallow and rapid breathing pattern. Moreover, airway smooth muscle cell (ASM) expansion is reduced, and the airway obstruction increases. Bronchial remodeling follows, further leading to airway hyperresponsiveness (AHR) and reduced expiratory reserve volume (ERV) and functional residual capacity (FRC) [31]. Both the forced vital capacity (FVC) and forced expiratory volume in 1 s (FEV1) are usually decreased in individuals with asthma and obesity [12]. Few studies have estimated these lung volumes in pediatric populations, and their reduction has been reported to be associated with high a body mass index (BMI). A retrospective study of 327 participants aged 6 to 17 years showed an association between increased BMI and lower lung volume measurements in spirometry, more specifically, FRC and RV values (*p* < 0.001). In the same study, the FEV1/FVC ratio decreased with an increasing BMI in both males and females (*p* < 0.001) [13]. In a cohort of 168 Hispanic and African American adolescents aged 13–18 years, trunk obesity was an independent predictor of ERV [32].

#### 3.1.2. Hormonal Milieu—Inflammation

Patients with obesity and asthma are also characterized by a non-atopic inflammation which is accentuated by hypoxia of the adipose tissue. In this state, leptin is secreted, M1 macrophages are activated, and CD4+ T lymphocytes differentiate into T-helper 1 (Th1) lymphocytes, with an increase in IL-6, TNF-α, IFN-γ, and interferon-γ–inducible protein (IP-1) [33,34]. Moreover, decreased levels of adiponectin are observed. This hormone normally stimulates the expression of the anti-inflammatory cytokine Interleukin-10 (IL-10) and Interleukin-1 (IL-1) receptor antagonists; thus its decrease enhances inflammation [34]. While most studies focus on adults, Nagel et al. conducted a study comprising 426 10-year-old children and noticed that low adiponectin levels and elevated leptin levels were linked with non-atopic asthma [35]. Another study of 85 children aged 6–10 years correlated low levels of adiponectin and increased levels of leptin with bronchial hyperresponsiveness after an exercise challenge in asthmatic patients [36]. Regarding the inflammatory process, Pacifico et al. correlated the Th1 immune profile and especially levels of IFN-γ with obesity and mainly with non-alcoholic steatohepatitis and insulin resistance [37]. In another study, high levels of IFN-γ were associated with high levels of leptin in asthmatic children with obesity, but not in asthmatic children with normal weight. Moreover, the former group showed worse FEV1 levels in spirometry, suggesting that this immune activation may play an important role in lung function [38]. Rastogi et al. associated IFN-γ levels with a low FEV1/FVC ratio in obese or overweight asthmatic pediatric patients [39]. Further, in a more recent study, the authors associated the Th-1 inflammation induced by insulin resistance in obese asthmatic adolescents with impaired lung function [40]. Although these studies have linked leptin and cytokine levels to asthma and pulmonary function in children with obesity, confirming that they aggravate the already existing asthma in such children, they cannot yet prove that asthma is a result of the inflammatory state that characterizes obesity [32].

### 3.2. Extrinsic Factors

#### 3.2.1. Sedentary Lifestyle and Activity

Obesity through inflammatory pathways may contribute to asthma risk in pediatric patients. Daily habits and activities have been studied in an attempt to identify the link between asthma and obesity. In a longitudinal study of children who had no asthma symptoms at 3.5 years of age, an increased time spent watching television was associated with asthma symptoms by 11.5 years [41]. Additionally, in a similar study of 757 participants with a median age of 9.7 years, reduced physical activity was weakly associated with an increased incidence of asthma during adolescence, suggesting that physical inactivity may be a risk factor for developing asthma [42]. In a study where all participants wore motion sensors, children who experienced wheezing episodes or were diagnosed with asthma within the past 12 months appeared to limit their physical activity, and especially sustained physical effort [43]. However, the authors did not determine whether asthma reduced activity or if a sedentary life leads to more frequent respiratory symptoms.

On the contrary, suboptimal asthma control in urban patients aged 7–9 years is associated with less moderate to vigorous physical activity (MVPA) and a higher BMI [44]. In another study, asthma appeared to be an inhibiting factor for sporting activities in pediatric patients aged 7–14 years old, and children with asthma had higher BMI levels, suggesting that asthma may be a predisposing factor for the development of obesity [45]. Similar were the findings in another study comprising adolescent girls with asthma [46]. A pooled analysis published in 2018 concluded that children diagnosed with asthma are more likely to develop obesity, with the association being particularly strong in those with active asthma (adjusted hazard ratio [aHR] 1.98) [47]. A systematic review published in 2020 by Li-Shen Shan et al. confirmed the bidirectional relationship between the two clinical entities [48]. Further, medications used by asthmatics, like inhaled corticosteroids, have been considered a contributing factor to obesity. An annual increase in ΒΜΙ was observed in patients 1 to 12 years of age on a high dose (>400 μg/day) inhaled corticosteroids compared with those receiving lower doses [49]. In addition, children with severe asthma and frequent inhaler use often avoid physical activity [47].

#### 3.2.2. Westernized Inflammatory Diet

A sedentary lifestyle in combination with the Western type of diet are key factors in the increase in obesity [50]. Furthermore, many studies have shown that this type of diet is associated with the increased risk of asthma and wheezing in children [51]. Omega-6 fatty acids in margarine and vegetable oils, with the most common being linoleic acid, which is converted into arachidonic acid and subsequently metabolized into Prostaglandin E**2** (PGE**2**), may lead to inflammation. Increased levels of Toll-Like Receptors (TLR) and pro-inflammatory cytokines (IL-6 and TNF-α) were observed in human studies after high fat and high carbohydrate administration [52,53]. Consumption of a fatty meal can elevate neutrophilic inflammation in the airways of patients with asthma and reduce the responsiveness of bronchodilators [52].

**Nutrition as a core intervention in the management of pediatric obesity and asthma and the role of the Mediterranean diet**

Diet is a key component in the management of obesity in children and adolescents. The American Academy of Pediatrics (AAP), along with European guidelines such as those from the National Institute for Health and Care Excellence (NICE) and the Italian Societies, emphasize the importance of dietary patterns and nutritional counseling by trained professionals for obesity management [54,55]. The consensus position statement by the Italian Society of Pediatric Endocrinology and Diabetology, the Italian Society of Pediatrics, and the Italian Society of Pediatric Surgery specifically recommends the MD as part of the management strategy for pediatric obesity, based on expert opinion and current best practices [56].

Recently, Abu-Qiyas et al. (2025) have reported that the vast majority of practicing dietitians view important components of the Mediterranean Diet—such as olive oil as the predominant source of fat and a greater intake of legumes and whole grains—as being both acceptable and feasible in pediatric nutritional counseling [57].

According to the Pavlidou et al. review, MD adoption has encouraging results on obesity, glycemic control, and cardiovascular risk, and is more beneficial compared to other diets in terms of its long-term effects [26].

Regarding asthma, several position papers, including those from the European Academy of Allergy and Clinical Immunology (EAACI), support the role of dietary diversity, particularly in the prevention or improved management of difficult-to-treat asthma [58]. However, they do not specifically recommend the MD, citing a lack of interventional evidence.

In contrast, the European Respiratory Society (ERS) highlights the growing importance of nutrition in asthma management, referencing the observational study by Papamichael et al., which suggests a potential protective role of the MD in childhood asthma. However, it highlights the lack of interventional studies, that could provide more reliable results [59,60].

Multiple benefits of the components of the MD are discussed below, suggesting that an emphasis on the Mediterranean dietary pattern could be part of the management of both diseases.

**Effects of the Mediterranean diet on weight management and metabolic health**

### 3.3. Mediterranean Diet: History and Components’ Benefits

The MD reflects the diet habits of inhabitants of countries around the Mediterranean Sea, especially Greece and Italy [27]. Since 1960 when it was first described, the MD has been globally studied by experts who focused on its components as important factors positively affecting people’s lifestyle, chronic diseases, lifespan, but also cancer risk. On November 2010, the Intergovernmental Committee of the UNESCO Convention approved the registration of the MD in the List of Intangible Cultural Heritage [27]. The MD pattern includes the daily consumption of vegetables, fruits, leafy plants, olive oil, whole grains, and a moderate intake of dairy products, followed by the weekly consumption of fish, poultry, legumes, and nuts and seeds and a decreased intake of red meat and sugary foods. In recent years, the above nutritional groups are depicted according to their frequency and quality in the “pyramid of the Mediterranean diet”.

Virgin olive oil, which is considered a symbol of the MD, is the main source of Monounsaturated Fatty Acids (MUFAs) like oleic acid. It also contains components of the unsaponifiable fraction, such as squalene and carotenoids, along with polyphenols, vitamins, and other micronutrients [61]. MUFAs, and especially oleic acid (n-9), have a protective role against inflammation by reducing inflammatory cytokines’ action and regulating the expression of genes involved in the inflammation cascade. Further, their beneficial effect on the cardiovascular system has been proven [62,63]. Polyphenols, found primarily in olive oil, vegetables, and fruits, are known for their impact on low-grade inflammation by decreasing cytokine production and their antioxidant action, contributing to the prevention of chronic diseases [64]. Several in vitro studies support the antimicrobial and antiviral actions of hydroxytyrosol, a polyphenol primarily found in olive oil, against various pathogens. These include microbes that affect the gut, such as Salmonella enterica and Yersinia enterocolitica, as well as microbes and viruses affecting the respiratory tract, including Haemophilus influenzae, SARS-CoV-2, and Influenza A [19,30,65]. Additionally, the antioxidant activity and the favorable effects on the cardiovascular system and cholesterol reduction in nutrients like vitamin C and vitamin E, found in many fruits and vegetables like tomatoes, have also been proven [66]. Fruits and vegetables are also a great source of glutathione peroxidase, vitamin A, and flavonoids known for their antioxidant and immune-modulating action [67]. Due to the high amount of fiber they contain, they also enhance satiety by slowing gastric emptying [25]. Further, the intestinal microbiome is strengthened through fiber fermentation, which regulates cytokine expression [68]. Short-chain fatty acids (SCFAs), like butyrate, are produced during fiber fermentation and have systemic anti-inflammatory effects by regulating T cell activity and promoting IL-10 secretion. Fiber is also rich in micronutrients and polyphenols, also characterized by an anti-inflammatory effect [69]. Selenium, a component of glutathione peroxidase, is an important element in many vegetables, animal products, and seafood in the MD such as tuna, salmon, oysters, shrimps, and sardines [70]. n-3 polyunsaturated fatty acids (PUFAs), found in oily fish such as salmon, mackerel, and sardines but also in leafy plants and some dairy products, are well known for their anti-inflammatory effect, as opposed to the n-6 PUFAS that enhance the inflammation cascade. More specifically, the omega-3 fatty acids, Linolenic Acid (LA), Eicosapentaenoic Acid (EPA), and Docosahexaenoic Acid (DHA) block the production of pro-inflammatory eicosanoids by acting initially on the enzyme delta-6 desaturase that inhibits the production of arachidonic acid from linoleic acid [71]. Another action is that they reduce the production of cyclooxygenase (COX), thereby inhibiting the metabolism of arachidonic acid [63].

### 3.4. Mediterranean Diet and Body Weight

As mentioned above, the MD may promote better body weight management due to mechanisms like the lower caloric density of its components and the enhanced feeling of satiety, the improved blood sugar regulation following vegetable and fish consumption, and the anti-inflammatory state due to fiber, polyphenols, omega-3 fatty acids, included also in fruits and olive oil.

In recent years, adherence to the MD has been extensively studied and successfully associated with low obesity rates in studies comparing BMI according to children’s MD compliance [21,22,72]. A recent cross-sectional study by Pavlidou et al. comprised 5188 preschool children from different regions of Greece and demonstrated that a better adherence to the MD was independently associated with lower obesity rates. Children with moderate or poor adherence had much higher rates of an increased BMI (*p* = 0.0024) [73].

Previous systematic reviews by Teixeira et al., Iaccarino et al., and Lassale et al., the latest of which was 2022, did not result in a clear correlation [74,75,76]. We isolated prospective studies described in the above reviews that focus on adherence to the Mediterranean diet and the BMI of pediatric patients after a period of follow-up (Table 1). Only English language studies were included. Eight studies detected a significant association between the adherence to MD and a decrease in BMI levels. Three other studies reported no significant improvement in BMI levels. A large study by Tognon et al., including 16,220 participants, found a statistically significant decrease in BMI after dietary intervention. McCourt’s study, which lasted 10 years, is the most extensive among those reviewed, yet it did not observe substantial changes in the BMI over the long term. This indicates that while short-term improvements in BMI may occur with adherence to the Mediterranean diet, the long-term maintenance of weight loss may be more challenging, especially in children and adolescents. Compliance with the Mediterranean diet in mentioned studies is calculated using scores from the international literature. The KIDMED Mediterranean Diet Quality Index for Children and Adolescents score is most widely used and is mainly in Mediterranean regions. Other scores used are the MDS (Mediterranean Diet Score), PREDIMED (Prevención con Dieta Mediterránea) Score, DASH (Dietary Approaches to Stop Hypertension), and DQI (Diet Quality Index) [74].

Certainly, maintaining one’s body mass index is a challenge, as many factors other than diet are involved such as genetic predisposition, lifestyle, and activities. Further, we observe that only a small number of prospective studies have been conducted in Mediterranean regions. Conducting such studies would be valuable, as they could account for potential homogeneity in lifestyle-related factors, which may influence the results.

### 3.5. Westernized Diet and Sedentary Lifestyle

Adopting the MD in everyday life is important in order to reduce the consumption of soft drinks, sweets, and junk food, which are primary elements of the Western diet. Fast foods contain cholesterol, sugar, refined carbohydrates, additives, trans fatty acids, and high proportions of saturated fat [52]. These fats activate Toll-Like Receptors (TLR), nuclear factor-kB (NF-kB) signaling, and induce the inflammatory response with the activation of IL-6, IL-16, IL-18, IL-8, TNF-a, MCP, and IFN-γ [52,85]. The C-Reactive Protein (CRP) is also elevated and associated with diseases like Type 2 Diabetes (T2D). In addition, adipocyte hypertrophy and triglyceride accumulation, enhanced by a Western diet and sedentary lifestyle, increases inflammation and worsens the metabolic profile of individuals [85]. In three related studies, the percentage of the daily caloric intake from processed products was calculated, showing a positive correlation with overweight and obesity rates. [86]. Sugar Sweetened Beverages (SSB), which contain the highest proportion of added sugars, seem to have a positive link with obesity in another review of 16 prospective studies, including 56,340 children [87]. Regarding sedentary lifestyle, in an observational study of 600 children over 5 years, the amount of time a child spends in a sedentary pattern is positively associated with the degree of obesity. In contrast, moderate-to-vigorous physical activity (MVPA) was negatively associated with BMI [88]. Overall, a reduced adherence to the MD was associated with overweight and obesity in children. Indeed, in a cross-sectional study from Greece that included not only dietary habits but also sleep duration and physical activity, summarized in a MediLIFE score, authors concluded that higher scores were associated with a lower BMI, as well as a lower prevalence of overweight, obesity, and abdominal obesity [89].

**Antioxidant and anti-inflammatory effects of MD and asthma**

### 3.6. Antioxidant-Rich Foods

According to the antioxidant hypothesis, the Western diet provides inadequate amounts of antioxidants, primarily sourced from fruits and vegetables [90]. Low intake of vegetables and grains in combination with a high sugar intake is considered a risk factor for asthma symptoms [34]. On the contrary, the MD is believed to contain components with antioxidant and anti-inflammatory traits. Vitamin C promotes airway hydration and neutralizes oxygen free radicals, while it also may modulate arachidonic acid release by affecting phospholipase A2 activity [91,92]. Additionally, it boosts the antioxidant effects of vitamin E. Isoforms of vitamin E are also linked to enhanced lung function [66]. Furthermore, higher carotenoid levels have been associated with improved respiratory function [93]. Findings from the Prevention and Incidence of Asthma and Mite Allergy (PIAMA) cohort, involving 4146 participants with up to 8 years of follow-up, indicated that the sustained consumption of fruits was negatively associated with the occurrence of asthma symptoms [24]. Fruit intake more than three times a week has a protective role in severe asthma for children and adolescents, in contrast with fast food intake, where the risk is elevated based on the International Study of Asthma and Allergies in Childhood (ISAAC)- Phase Three [94].

Vitamin E, a fat-soluble antioxidant contained in vegetables, eggs, and seed oils, impedes lipid peroxidation and, consequently, protects cell membranes from oxidation [95]. By reducing endogenous antioxidants, vitamin E is associated with a reduction in the production of inflammatory mediators such as TNF-α, IL-1, and IL-8 through the modulation of NF-κB signaling [96]. However, various vitamin E isoforms, named tocopherols, seem to have opposing effects on lung function, as α-tocopherol has a protective effect on asthma control while γ-tocopherol seems to aggravate asthma attacks [97]. In an interventional study comprising 240 children, 2–17 years of age, treatment with vitamin E improved the FEV1/FVC ratio in spirometry, while supplementation with vitamin E in adults with asthma showed no clear benefit [98]. Prenatal vitamin E levels also seem to play an important role, since maternal vitamin E intake during pregnancy was negatively associated with an asthma diagnosis in the first 10 years (OR 0.89, 95% CI 0.81–0.99) [99]. In a study that recorded the dietary habits of a pediatric population, wheezing and asthma symptoms were associated with a reduced intake of micronutrients such as vitamin E, but the association with vitamin C or selenium was not clear [100]. Quantitatively, vitamin C and tocopherol levels did not differ between the two groups of Caucasian children with and without asthma [101].

As for vitamin C or Ascorbic acid, it is a water-soluble component, especially found in citrus fruits such as oranges and lemons. In addition to its effect on the absorption of other nutrients, it is also an antioxidant found on the airway surface liquid (ASL) of the lungs [102]. When 18,737 children, aged 6–7 years, from Italy were surveyed about their dietary habits related to vitamin C-containing fruits, an increased intake was associated with a reduction in asthma and wheezing exacerbations, especially in children with such a predisposition [103]. According to a Cochrane systematic review, Vitamin C supplementation may improve asthma control scores and FEV1 values in children with asthma, aged 7–8 years, when compared to a placebo [104]. In addition, in a study comprising a small population of adolescents with exercise-induced bronchoconstriction, a modest improvement in symptoms after vitamin C supplementation was demonstrated [105].

### 3.7. Inflammatory Foods

Avoidance of high-fat foods is very important, as they enhance inflammation [52]. An early study of adults showed that a high margarine intake was associated with asthma development [106]. Trans fatty acids found in hydrogenated oils, like margarine, have also been associated with asthma and atopy. Therefore, the decreased consumption of such fatty acids that characterize the MD could improve the overall metabolic profile of individuals with obesity and asthma. A recent review conducted by Wang et al., related fast food intake, especially hamburgers, with asthma and current wheeze in a dose-dependent way [107]. Concerning soft drinks, several studies have associated their consumption with asthma in pediatric populations. Sweetened soft drinks containing sodium benzoate or potassium have been directly associated with asthma. Their increased consumption is also associated with the incidence of obesity, which additionally facilitates the development of asthma symptoms [108]. Furthermore, fructose corn syrup consumption, which has increased considerably in Western diets during the last decade, has been associated with hypersecretion of mucus from the airways [108].

### 3.8. Omega-3 Fatty Acids

Regarding healthy fats and their anti-inflammatory effects, several studies have explored their correlation with normal lung function. Supplementing pregnant women with long-chain fatty acids (LCFAs), starting from the 24th week of gestation, has been linked to fewer asthma symptoms in their children at ages 3 and 5, particularly when derived from fish oil [109]. Additionally, infants who received formula milk supplemented with LCFAs from <6 months through 12 months of age were found to have a lower risk of asthma and respiratory infections by age 3 [110]. In a study that included a questionnaire answered by 574 children and their parents about fish consumption, a higher frequency of fish intake was associated with reduced asthma symptoms [111]. Based on a survey conducted in six European countries with 20,271 pediatric participants, a low intake of fish independently predicted a persistent cough (OR = 1.14; 95% CI 1.03 to 1.25), wheeze in general (OR = 1.14; 95% CI 1.03 to 1.25), and current wheeze (OR = 1.21; 95% CI 1.06 to 1.39) [112]. However, some observational studies did not demonstrate any association of n-3/n-6 fatty acids with asthma and atopy. Similarly, interventional studies did not show a notable reduction in the frequency of respiratory symptoms after supplementation with omega-3 fatty acids [113]. To obtain more reliable results, it is crucial to conduct larger and longer interventional studies, which will track individuals from fetal life through late childhood, adolescence, and early adult life.

### 3.9. Vitamin D

Vitamin D, a steroid hormone acquired through diet to some extent but mainly synthesized in the skin via sun exposure, plays a crucial role in health [114]. Low levels of vitamin D have been associated with both asthma and obesity [114]. A deficiency in vitamin D can lead to the expression of TLRs on macrophages, resulting in the production of pro-inflammatory cytokines [115]. Additionally, vitamin D receptors (VDR) in airway smooth muscle cells may exacerbate asthma symptoms in individuals with a deficiency by suppressing cytokine expression [115]. In the context of obesity, it is speculated that a sedentary lifestyle among patients with obesity leads to reduced sun exposure, contributing to lower vitamin D levels. Moreover, vitamin D is stored in adipocytes, reducing its bioavailability in obese individuals [116]. Studies in both adults and children have shown that low vitamin D levels are associated with poorer lung function, as indicated by spirometry results [116]. Notably, an interventional study involving 48 pediatric patients demonstrated a decrease in asthma flare-ups following infections in children who received vitamin D supplements [117]. Similarly, vitamin D supplementation in winter has been linked to reduced rates of Influenza A infections and thus asthma exacerbations [118].

## 4. Limitations and Research Gaps

The narrative review has several limitations. The available studies are heterogeneous in population, design, and outcome measures, which makes comparisons difficult and weakens the conclusions. Another potential limitation is the chance of publication bias, as negative or null findings may be underrepresented. Additionally, most of the evidence so far comes from observational studies. Large-scale interventional trials specifically addressing the MD in pediatric obesity and asthma remain scarce. Standardized longitudinal outcome research is still lacking, and the potential impact of confounding lifestyle and socioeconomic determinants is insufficiently explored. Moreover, comparison with other dietary patterns could further clarify the independent contribution of the MD. Finally, studies evaluating how frequently pediatricians recommend the MD in daily clinical practice are missing, highlighting an important gap in implementation research. Therefore, the findings should be interpreted with caution. Future research needs to strengthen observational evidence using standardized methods, expand longitudinal and interventional studies, and address clinical practice aspects to help draw causal inference.

## 5. Conclusions

The rise in pediatric obesity and asthma in recent decades has become a significant public health issue that could negatively affect the health of generations to come. Several pathomechanisms have been identified to play an interconnected role in the pathogenesis of the two diseases. The MD has been shown to not only improve the metabolic status in the general population but also to contribute to the curtailment and improvement of the main components and complications of obesity and asthma both in adults and in children. Therefore, the MD as a component of a comprehensive public health strategy approach, including increased physical activity, decreased screen time, and proper sleep, could contribute to weight management and a reduction in asthma symptoms with the ultimate goal of fewer complications and improved overall health.

Future directions should be toward the systematic evaluation of Mediterranean dietary modules in childhood obesity and asthma through large-scale, long-term research, with a particular emphasis on optimizing adherence and effect measurements. In addition, approaches to sensitization and the education of families and children to the MD and the routine incorporation of use of validated tools like the KIDMED score for food monitoring in clinical and public health practice can enhance prevention and control.

## Figures and Tables

**Table 1:** Prospective studies focusing on adherence to MD and the association with BMI in children and adolescents. Abbreviations: KIDMED, Mediterranean Diet Quality Index for Children and Adolescents; DASH, Dietary Approaches to Stop Hypertension; DQI, Diet Quality Index; MDS, Mediterranean Diet Score; OHS, Oslo Health Study; rMED, Relative Mediterranean Diet Score; fMDS, Food Frequency-based Mediterranean Diet Score.

Author	Participants	Date	Ages (Years Old)	Duration of Follow-Up	Assessment Methods	Result
Asghari et al., Iran [77]	424	2016	6–18	3.6 years	DASH	No significant decreasing trend (*p* = 0.192)
Lioret et al., Australia [78]	216	2014	5–12	3 years	DQI	Score inversely related to BMI in overweight children at Baseline; (*p* = 0.078)
Martin-Calvo et al., United States [79]	10,918	2016	8–15	2–3 years	KIDMED	2-point increment in the index is associated −0.04 kg/m^2^; *p* = 0.001
McCourt et al., Ireland [80]	487	2014	12–15	10 years	MDS	No association
Monjardino et al., Portugal [81]	1716	2012	13	4 years	DASH, OHS	No significant association
Notario et al., United States [72]	1527	2020	4	4 years	rMED	Score inversely associated (*p* = 0.02)
Ojeda-Rodríguez et al., Pamplona [82]	107	2018	10	2 years	KIDMED	Inversely associated
Ranucci et al., Perugia [83]	74	2017	5–17	3 months	KIDMED	Inversely associated in children group; *p* < 0.01
Tognon et al., 8 European countries [84]	16.220	2014	2–9	2 years	fMDS	High fMDS scores were inversely associated with overweight, obesity (*p* = 0.001)

## References

[1] Di Cesare M., Sorić M., Bovet P., Miranda J.J., Bhutta Z., Stevens G.A., Laxmaiah A., Kengne A.-P., Bentham J. (2019). The Epidemiological Burden of Obesity in Childhood: A Worldwide Epidemic Requiring Urgent Action. BMC Med..

[2] Uphoff E.P., Bird P.K., Antó J.M., Basterrechea M., von Berg A., Bergström A., Bousquet J., Chatzi L., Fantini M.P., Ferrero A. (2017). Variations in the Prevalence of Childhood Asthma and Wheeze in MeDALL Cohorts in Europe. ERJ Open Res..

[3] Pearce N., Aït-Khaled N., Beasley R., Mallol J., Keil U., Mitchell E.A., Robertson C., ISAAC Phase Three Study Group (2007). Worldwide Trends in the Prevalence of Asthma Symptoms: Phase III of the International Study of Asthma and Allergies in Childhood (ISAAC). Thorax.

[4] García-Marcos L., Asher M.I., Pearce N., Ellwood E., Bissell K., Chiang C.Y., El Sony A., Ellwood P., Marks G.B., Mortimer K. (2022). The Burden of Asthma, Hay Fever and Eczema in Children in 25 Countries: GAN Phase I Study. Eur. Respir. J..

[5] NCD Risk Factor Collaboration (NCD-RisC) (2017). Worldwide Trends in Body-Mass Index, Underweight, Overweight, and Obesity from 1975 to 2016: A Pooled Analysis of 2416 Population-Based Measurement Studies in 128.9 Million Children, Adolescents, and Adults. Lancet.

[6] Parasuaraman G., Ayyasamy L., Aune D., Sen A., Nagarajan R., Rajkumar P., Velusamy S., Manickam P., Sivaprakasam S. (2023). The Association between Body Mass Index, Abdominal Fatness, and Weight Change and the Risk of Adult Asthma: A Systematic Review and Meta-Analysis of Cohort Studies. Sci. Rep..

[7] Lang J.E., Bunnell H.T., Hossain J., Wysocki T., Lima J.J., Finkel T.H., Bacharier L., Dempsey A., Sarzynski L., Test M. (2018). Being Overweight or Obese and the Development of Asthma. Pediatrics.

[8] Ahmadizar F., Vijverberg S.J.H., Arets H.G.M., de Boer A., Lang J.E., Kattan M., Palmer C.N.A., Mukhopadhyay S., Turner S., Maitland-van der Zee A.H. (2016). Childhood Obesity in Relation to Poor Asthma Control and Exacerbation: A Meta-Analysis. Eur. Respir. J..

[9] Deng X., Ma J., Yuan Y., Zhang Z., Niu W. (2019). Association between Overweight or Obesity and the Risk for Childhood Asthma and Wheeze: An Updated Meta-Analysis. Pediatr. Obes..

[10] Chen Z., Salam M.T., Alderete T.L., Habre R., Bastain T.M., Berhane K., Gilliland F.D. (2017). Effects of Childhood Asthma on the Development of Obesity among School-Aged Children. Am. J. Respir. Crit. Care Med..

[11] Garcia-Rio F., Alvarez-Puebla M.J., Esteban-Gorgojo I., Barranco P., Olaguibel J.M. (2019). Obesity and Asthma: Key Clinical Questions. J. Investig. Allergol. Clin. Immunol..

[12] Beuther D.A., Weiss S.T., Sutherland E.R. (2006). Obesity and Asthma. Am. J. Respir. Crit. Care Med..

[13] Davidson W.J., Mackenzie-Rife K.A., Witmans M.B., Montgomery M.D., Ball G.D.C., Egbogah S., Faulkner G., McMorris C., Poulin P., Thabane L. (2014). Obesity Negatively Impacts Lung Function in Children and Adolescents. Pediatr. Pulmonol..

[14] Bantulà M., Roca-Ferrer J., Arismendi E., Picado C. (2021). Asthma and Obesity: Two Diseases on the Rise and Bridged by Inflammation. J. Clin. Med..

[15] Peters M.C., McGrath K.W., Hawkins G.A., Hastie A.T., Levy B.D., Israel E., Phillips B.R., Mauger D.T., Ortega V.E., Coverstone A. (2016). Plasma Interleukin-6 Concentrations, Metabolic Dysfunction, and Asthma Severity: A Cross-Sectional Analysis. Lancet Respir. Med..

[16] Osman A.M.E., Motawie A.A.M., Abd Al-Aziz A.M., Mostafa N.A.A., Hasan N.S., El-Baz M.S. (2023). Role of Adiponectin, Resistin and Monocyte Chemo-Attractant Protein-1 in Overweight/Obese Asthma Phenotype in Children. BMC Pediatr..

[17] Arshi M., Cardinal J., Hill R.J., Davies P.S.W., Wainwright C. (2010). Asthma and Insulin Resistance in Children. Respirology.

[18] Al-Shawwa B.A., Al-Huniti N.H., DeMattia L., Gershan W. (2007). Asthma and Insulin Resistance in Morbidly Obese Children and Adolescents. J. Asthma.

[19] Sood A., Cui X., Qualls C., Beckett W.S., Gross M.D., Steffes M.W., Smith L.J., Jacobs D.R. (2008). Association between Asthma and Serum Adiponectin Concentration in Women. Thorax.

[20] Sood A., Shore S.A. (2013). Adiponectin, Leptin, and Resistin in Asthma: Basic Mechanisms through Population Studies. J. Allergy.

[21] López-Gil J.F., García-Hermoso A., Sotos-Prieto M., Cavero-Redondo I., Martínez-Vizcaíno V., Kales S.N. (2023). Mediterranean Diet-Based Interventions to Improve Anthropometric and Obesity Indicators in Children and Adolescents: A Systematic Review with Meta-Analysis. Adv. Nutr..

[22] Kanellopoulou A., Giannakopoulou S.P., Notara V., Antonogeorgos G., Rojas-Gil A.P., Kornilaki E.N., Konstantinou E., Lagiou A., Panagiotakos D.B. (2021). The Association between Adherence to the Mediterranean Diet and Childhood Obesity; the Role of Family Structure. Nutr. Health.

[23] Koumpagioti D., Boutopoulou B., Moriki D., Priftis K.N., Douros K. (2022). Does Adherence to the Mediterranean Diet Have a Protective Effect against Asthma and Allergies in Children? A Systematic Review. Nutrients.

[24] Willers S.M., Wijga A.H., Brunekreef B., Scholtens S., Postma D.S., Kerkhof M., Gerritsen J., de Jongste J.C., Smit H.A. (2011). Childhood Diet and Asthma and Atopy at 8 Years of Age: The PIAMA Birth Cohort Study. Eur. Respir. J..

[25] Douros K., Thanopoulou M.I., Boutopoulou B., Papadopoulou A., Papadimitriou A., Fretzayas A., Tsabouri S., Nicolaidou P., Priftis K.N. (2019). Adherence to the Mediterranean Diet and Inflammatory Markers in Children with Asthma. Allergol. Immunopathol..

[26] Pavlidou E., Papadopoulou S.K., Fasoulas A., Papaliagkas V., Alexatou O., Chatzidimitriou M., Tsaliki M., Petridis D., Tzimos C., Tsonidis C. (2024). Diabesity and Dietary Interventions: Evaluating the Impact of Mediterranean Diet and Other Types of Diets on Obesity and Type 2 Diabetes Management. Nutrients.

[27] Kiani A.K., Medori M.C., Bonetti G., Aquilanti B., Velluti V., Matera G., Stuppia L., Puca A.A., Romeo G., Colonna V. (2022). Modern Vision of the Mediterranean Diet. J. Prev. Med. Hyg..

[28] Shah Gupta R., Koteci A., Morgan A., George P.M., Quint J.K. (2023). Incidence and Prevalence of Interstitial Lung Diseases Worldwide: A Systematic Literature Review. BMJ Open Respir. Res..

[29] Schatz M., Hsu J.W.Y., Zeiger R.S., Chen W., Dorenbaum A., Chipps B.E., Haselkorn T., Borish L., Weiss S.T., Peters S.P. (2014). Phenotypes Determined by Cluster Analysis in Severe or Difficult-to-Treat Asthma. J. Allergy Clin. Immunol..

[30] Ali Z., Ulrik C.S. (2013). Obesity and Asthma: A Coincidence or a Causal Relationship? A Systematic Review. Respir. Med..

[31] Sansone F., Attanasi M., Di Pillo S., Chiarelli F. (2020). Asthma and Obesity in Children. Biomedicines.

[32] Rastogi D., Bhalani K., Hall C.B., Isasi C.R. (2014). Association of Pulmonary Function with Adiposity and Metabolic Abnormalities in Urban Minority Adolescents. Ann. Am. Thorac. Soc..

[33] Vijayakanthi N., Greally J.M., Rastogi D. (2016). Pediatric Obesity-Related Asthma: The Role of Metabolic Dysregulation. Pediatrics.

[34] Shore S.A., Johnston R.A. (2006). Obesity and Asthma. Pharmacol. Ther..

[35] Nagel G., Koenig W., Rapp K., Wabitsch M., Zoellner I., Weiland S.K. (2009). Associations of Adipokines with Asthma, Rhinoconjunctivitis, and Eczema in German Schoolchildren. Pediatr. Allergy Immunol..

[36] Baek H.S., Kim Y.D., Shin J.H., Kim J.H., Oh J.W., Lee H.B. (2011). Serum Leptin and Adiponectin Levels Correlate with Exercise-Induced Bronchoconstriction in Children with Asthma. Ann. Allergy Asthma Immunol..

[37] Pacifico L., Di Renzo L., Anania C., Osborn J.F., Ippoliti F., Schiavo E., Chiesa C. (2006). Increased T-Helper Interferon-γ-Secreting Cells in Obese Children. Eur. J. Endocrinol..

[38] Youssef D., Mohamed Elbehidy R., Mahamoud Shokry D., Mohamed Elbehidy E. (2013). The Influence of Leptin on Th1/Th2 Balance in Obese Children with Asthma. J. Bras. Pneumol..

[39] Rastogi D., Canfield S.M., Andrade A., Isasi C.R., Hall C.B., Rubinstein A., Arens R., Chung K.F. (2012). Obesity-Associated Asthma in Children: A Distinct Entity. Chest.

[40] Rastogi D., Fraser S., Oh J., Huber A.M., Schulman Y., Bhagtani R.H., Khan Z.S., Tesfa L., Hall C.B., Brunner E. (2015). Inflammation, Metabolic Dysregulation, and Pulmonary Function among Obese Urban Adolescents with Asthma. Am. J. Respir. Crit. Care Med..

[41] Sherriff A., Maitra A., Ness A.R., Mattocks C., Riddoch C., Reilly J.J., Paton J.Y., Henderson A.J. (2009). Association of Duration of Television Viewing in Early Childhood with the Subsequent Development of Asthma. Thorax.

[42] Rasmussen F., Lambrechtsen J., Siersted H.C., Hansen H.S., Hansen N.C. (2000). Low Physical Fitness in Childhood Is Associated with the Development of Asthma in Young Adulthood: The Odense Schoolchild Study. Eur. Respir. J..

[43] Firrincieli V., Keller A., Ehrensberger R., Platts-Mills J., Shufflebarger C., Geldmaker B., Metayer C., Etzel R., Stigler S., Wagner L. (2005). Decreased Physical Activity among Head Start Children with a History of Wheezing: Use of an Accelerometer to Measure Activity. Pediatr. Pulmonol..

[44] Koinis-Mitchell D., Kopel S.J., Dunsiger S., McQuaid E.L., Miranda L.G., Mitchell P., Boergers J., Fritz G.K. (2021). Asthma and Physical Activity in Urban Children. J. Pediatr. Psychol..

[45] Glazebrook C., McPherson A.C., Macdonald I.A., Swift J.A., Ramsay C., Newbould R., Smyth A. (2006). Asthma as a Barrier to Children’s Physical Activity: Implications for Body Mass Index and Mental Health. Pediatrics.

[46] Groth S.W., Rhee H., Kitzman H. (2016). Relationships among obesity, physical activity and sedentary behavior in young adolescents with and without lifetime asthma. J. Asthma.

[47] Contreras Z.A., Chen Z., Roumeliotaki T., Annesi-Maesano I., Baïz N., von Berg A., Bauer C.P., Berdel D., Berhane K., Chatzi L. (2018). Does Early Onset Asthma Increase Childhood Obesity Risk? A Pooled Analysis of 16 European Cohorts. Eur. Respir. J..

[48] Shan L.S., Zhou Q.L., Shang Y.X. (2020). Bidirectional Association between Asthma and Obesity during Childhood and Adolescence: A Systematic Review and Meta-Analysis. Front. Pediatr..

[49] Jani M., Ogston S., Mukhopadhyay S. (2005). Annual Increase in Body Mass Index in Children with Asthma on Higher Doses of Inhaled Steroids. J. Pediatr..

[50] Verduci E., Bronsky J., Embleton N., Gerasimidis K., Indrio F., Köglmeier J., de Koning B., Mihatsch W., Vandenplas Y., van Goudoever J.B. (2021). Role of Dietary Factors, Food Habits, and Lifestyle in Childhood Obesity Development: A Position Paper from the European Society for Paediatric Gastroenterology, Hepatology and Nutrition Committee on Nutrition. J. Pediatr. Gastroenterol. Nutr..

[51] Patel S., Custovic A., Smith J.A., Simpson A., Kerry G., Murray C.S. (2014). Cross-Sectional Association of Dietary Patterns with Asthma and Atopic Sensitization in Childhood: A Cohort Study. Pediatr. Allergy Immunol..

[52] Wood L.G., Garg M.L., Gibson P.G. (2011). A High-Fat Challenge Increases Airway Inflammation and Impairs Bronchodilator Recovery in Asthma. J. Allergy Clin. Immunol..

[53] Ghanim H., Sia C.L., Upadhyay M., Korzeniewski K., Viswanathan P., Abuaysheh S., Mohanty P., Dandona P. (2010). Orange Juice Neutralizes the Proinflammatory Effect of a High-Fat, High-Carbohydrate Meal and Prevents Endotoxin Increase and Toll-Like Receptor Expression. Am. J. Clin. Nutr..

[54] NICE Physical Activity and Diet: Overweight and Obesity Management. NICE Guidance. https://www.nice.org.uk/guidance.

[55] Hampl S.E., Hassink S.G., Skinner A.C., Armstrong S.C., Barlow S.E., Bolling C.F., Daniels S.R., de Ferranti S.D., Golden N.H., Kelly A.S. (2023). Clinical Practice Guideline for the Evaluation and Treatment of Children and Adolescents with Obesity. Pediatrics.

[56] Maffeis C., Olivieri F., Valerio G., Verduci E., Licenziati M.R., Calcaterra V., Pietrobelli A., Miraglia Del Giudice E., Brufani C., Grugni G. (2023). The Treatment of Obesity in Children and Adolescents: Consensus Position Statement of the Italian Societies. Ital. J. Pediatr..

[57] Abu-Qiyas S., Radwan H., Cheikh Ismail L., Alameddine M., Muayyad M., Naja F. (2025). Knowledge, Attitudes, and Use of the Mediterranean Diet in Practice among Dietitians in the UAE. Sci. Rep..

[58] Venter C., Greenhawt M., Meyer R.W., Agostoni C., Reese I., du Toit G., Fleischer D.M., Maslin K., Nwaru B.I., Roduit C. (2020). EAACI Position Paper on Diet Diversity in Pregnancy, Infancy and Childhood. Allergy.

[59] Papamichael M.M., Itsiopoulos C., Susanto N.H., Erbas B. (2017). Does Adherence to the Mediterranean Dietary Pattern Reduce Asthma Symptoms in Children? A Systematic Review. Public Health Nutr..

[60] Stoodley I., Williams L., Thompson C., Scott H., Wood L. (2019). Evidence for Lifestyle Interventions in Asthma. Breathe.

[61] Piroddi M., Albini A., Fabiani R., Giovannelli L., Luceri C., Natella F., Santangelo C., Serafini M., Storniolo C.E., Visioli F. (2017). Nutrigenomics of Extra-Virgin Olive Oil: A Review. Biofactors.

[62] Mazzocchi A., Leone L., Agostoni C., Pali-Schöll I. (2019). The Secrets of the Mediterranean Diet: Does [Only] Olive Oil Matter?. Nutrients.

[63] Vassilopoulou E., Guibas G.V., Papadopoulos N.G. (2022). Mediterranean-Type Diets as a Protective Factor for Asthma and Atopy. Nutrients.

[64] Bonaccio M., Pounis G., Cerletti C., Donati M.B., Iacoviello L., de Gaetano G. (2017). Mediterranean Diet, Dietary Polyphenols and Low Grade Inflammation: Results from the MOLI-SANI Study. Br. J. Clin. Pharmacol..

[65] Marković A.K., Torić J., Barbarić M., Brala C.J. (2019). Hydroxytyrosol, Tyrosol and Derivatives and Their Potential Effects on Human Health. Molecules.

[66] Traber M.G., Atkinson J. (2007). Vitamin E, Antioxidant and Nothing More. Free Radic. Biol. Med..

[67] Bellik Y., Boukraâ L., Alzahrani H.A., Bakhotmah B.A., Abdellah F., Hammoudi S.M., Iguer-Ouada M. (2013). Molecular Mechanism Underlying Anti-Inflammatory and Anti-Allergic Activities of Phytochemicals: An Update. Molecules.

[68] Murga-Garrido S.M., Hong Q., Cross T.W.L., Hutchison E.R., Han J., Thomas S.P., Chen J., Carmody R.N., Gaskins H.R., Swanson K.S. (2021). Gut Microbiome Variation Modulates the Effects of Dietary Fiber on Host Metabolism. Microbiome.

[69] Sood A., Qualls C., Schuyler M., Thyagarajan B., Steffes M.W., Smith L.J., Jacobs D.R. (2012). Low Serum Adiponectin Predicts Future Risk for Asthma in Women. Am. J. Respir. Crit. Care Med..

[70] Guillin O.M., Vindry C., Ohlmann T., Chavatte L. (2019). Selenium, Selenoproteins and Viral Infection. Nutrients.

[71] Ishihara T., Yoshida M., Arita M. (2019). Omega-3 Fatty Acid-Derived Mediators That Control Inflammation and Tissue Homeostasis. Int. Immunol..

[72] Notario-Barandiaran L., Valera-Gran D., Gonzalez-Palacios S., Garcia-de-la-Hera M., Fernández-Barrés S., Pereda-Pereda E., Fernandez-Somoano A., Guxens M., Iniguez C., Romaguera D. (2020). High Adherence to a Mediterranean Diet at Age 4 Reduces Overweight, Obesity and Abdominal Obesity Incidence in Children at the Age of 8. Int. J. Obes..

[73] Pavlidou E., Papadopoulou S.K., Alexatou O., Voulgaridou G., Mentzelou M., Biskanaki F., Psara E., Tsourouflis G., Lefantzis N., Dimoliani S. (2023). Childhood Mediterranean Diet Adherence Is Associated with Lower Prevalence of Childhood Obesity, Specific Sociodemographic, and Lifestyle Factors: A Cross-Sectional Study in Pre-School Children. Epidemiologia.

[74] Teixeira B., Afonso C., Rodrigues S., Oliveira A. (2022). Healthy and Sustainable Dietary Patterns in Children and Adolescents: A Systematic Review. Adv. Nutr..

[75] Iaccarino Idelson P., Scalfi L., Valerio G. (2017). Adherence to the Mediterranean Diet in Children and Adolescents: A Systematic Review. Nutr. Metab. Cardiovasc. Dis..

[76] Lassale C., Fitó M., Morales-Suárez-Varela M., Moya A., Gómez S.F., Schröder H. (2022). Mediterranean Diet and Adiposity in Children and Adolescents: A Systematic Review. Obes. Rev..

[77] Asghari G., Yuzbashian E., Mirmiran P., Hooshmand F., Najafi R., Azizi F. (2016). DASH Dietary Pattern and Reduced Incidence of Metabolic Syndrome in Children and Adolescents. J. Pediatr..

[78] Lioret S., McNaughton S.A., Cameron A.J., Crawford D., Campbell K.J., Cleland V.J., Ball K. (2014). Three-Year Change in Diet Quality and Associated Changes in BMI among Schoolchildren Living in Socioeconomically Disadvantaged Neighbourhoods. Br. J. Nutr..

[79] Martin-Calvo N., Chavarro J.E., Falbe J., Hu F.B., Field A.E. (2016). Adherence to the Mediterranean Dietary Pattern and BMI Change among U.S. Adolescents. Int. J. Obes..

[80] McCourt H.J., Draffin C.R., Woodside J.V., Cardwell C.R., Young I.S., Hunter S.J., Murray L.J., Boreham C.A., Gallagher A.M., Neville C.E. (2014). Dietary Patterns and Cardiovascular Risk Factors in Adolescents and Young Adults: Northern Ireland Young Hearts Project. Br. J. Nutr..

[81] Monjardino T., Lucas R., Ramos E., Barros H. (2014). Associations between a Priori-Defined Dietary Patterns and Longitudinal Changes in Bone Mineral Density in Adolescents. Public Health Nutr..

[82] Ojeda-Rodríguez A., Zazpe I., Morell-Azanza L., Chueca M.J., Azcona-Sanjulián M.C., Marti A. (2018). Improved Diet Quality and Nutrient Adequacy after a Lifestyle Intervention in Children with Abdominal Obesity. Nutrients.

[83] Ranucci C., Pippi R., Buratta L., Aiello C., Gianfredi V., Piana N., Reginato E., Romano L., Lorenzoni V., Pippi R. (2017). Effects of an Intensive Lifestyle Intervention in Overweight/Obese Children and Adolescents. Biomed. Res. Int..

[84] Tognon G., Hebestreit A., Lanfer A., Moreno L.A., Pala V., Siani A., Veidebaum T., Tornaritis M., Molnár D., De Henauw S. (2014). Mediterranean Diet, Overweight and Body Composition in Children from Eight European Countries: IDEFICS Study. Nutr. Metab. Cardiovasc. Dis..

[85] Ravaut G., Légiot A., Bergeron K.F., Mounier C. (2021). Monounsaturated Fatty Acids in Obesity-Related Inflammation. Int. J. Mol. Sci..

[86] Poti J.M., Braga B., Qin B. (2017). Ultra-Processed Food Intake and Obesity: What Really Matters for Health—Processing or Nutrient Content?. Curr. Obes. Rep..

[87] Luger M., Lafontan M., Bes-Rastrollo M., Winzer E., Yumuk V., Farpour-Lambert N. (2018). Sugar-Sweetened Beverages and Weight Gain in Children and Adults: A Systematic Review from 2013 to 2015 and a Comparison with Previous Studies. Obes. Facts.

[88] Schwarzfischer P., Gruszfeld D., Socha P., Luque V., Closa-Monasterolo R., Rousseaux D., Moretti M., Mariani B., Verduci E., Koletzko B. (2018). Longitudinal Analysis of Physical Activity, Sedentary Behaviour and Anthropometry Ages 6 to 11 Years. Int. J. Behav. Nutr. Phys. Act..

[89] Katsagoni C.N., Psarra G., Georgoulis M., Tambalis K., Panagiotakos D.B., Sidossis L.S., EYZN Study Group (2020). High and Moderate Adherence to Mediterranean Lifestyle Is Inversely Associated with Overweight, General and Abdominal Obesity in Children and Adolescents: The MediLIFE-Index. Nutr. Res..

[90] Seaton A., Godden D.J., Brown K. (1994). Increase in Asthma: A More Toxic Environment or a More Susceptible Population?. Thorax.

[91] Padayatty S.J., Katz A., Wang Y., Eck P., Kwon O., Lee J.H., Chen S., Corpe C., Dutta A., Dutta S.K. (2003). Vitamin C as an Antioxidant: Role in Disease Prevention. J. Am. Coll. Nutr..

[92] Johnston S.L., Freezer N.J., Ritter W., O’Toole S., Howarth P.H. (1995). Prostaglandin D2-Induced Bronchoconstriction: Role of Thromboxane Prostanoid Receptor. Eur. Respir. J..

[93] Semba R.D., Chang S.S., Sun K., Talegawkar S., Ferrucci L., Fried L.P. (2012). Serum Carotenoids and Pulmonary Function in Older Community-Dwelling Women. J. Nutr. Health Aging.

[94] Ellwood P., Asher M.I., García-Marcos L., Williams H., Keil U., Robertson C., Nagel G., ISAAC Phase III Study Group (2013). Do fast foods cause asthma, rhinoconjunctivitis and eczema? Global findings from the International Study of Asthma and Allergies in Childhood (ISAAC) Phase Three. Thorax.

[95] Romieu I., Trenga C. (2001). Diet and Obstructive Lung Diseases. Epidemiol. Rev..

[96] Shams M.H., Jafari R., Eskandari N., Masjedi M., Kheirandish F., Ganjalikhani Hakemi M., Ahmadi M., Kalantari H., Rezaei N. (2021). Anti-Allergic Effects of Vitamin E in Allergic Diseases: An Updated Review. Int. Immunopharmacol..

[97] Cook-Mills J.M. (2013). Isoforms of Vitamin E Differentially Regulate PKCα and Inflammation: A Review. J. Clin. Cell. Immunol..

[98] Ghaffari J., Hossaini R.F., Khalilian A., Nahanmoghadam N., Salehifar E., Rafatpanah H. (2014). Vitamin E Supplementation, Lung Functions and Clinical Manifestations in Children with Moderate Asthma: A Randomized Double-Blind Placebo-Controlled Trial. Iran. J. Allergy Asthma Immunol..

[99] Allan K.M., Prabhu N., Craig L.C.A., McNeill G., Kirby B., McLay J., Helms P.J., Ayres J.G., Seaton A., Turner S.W. (2015). Maternal Vitamin D and E Intakes during Pregnancy and Childhood Asthma. Eur. Respir. J..

[100] Hijazi N., Abalkhail B., Seaton A. (2000). Diet and Childhood Asthma in a Society in Transition: A Study in Urban and Rural Saudi Arabia. Thorax.

[101] Powell C.V.E., Nash A.A., Powers H.J., Primhak R.A. (1994). Antioxidant Status in Asthma. Pediatr. Pulmonol..

[102] Zajac D., Wojciechowski P. (2023). The Role of Vitamins in the Pathogenesis of Asthma. Int. J. Mol. Sci..

[103] Forastiere F., Pistelli R., Sestini P., Fortes C., Renzoni E., Rusconi F., Dell’Orco V., Ciccone G., Bisanti L., SIDRIA Collaborative Group (2000). Consumption of Fresh Fruit Rich in Vitamin C and Wheezing Symptoms in Children. Thorax.

[104] Kaur B., Rowe B.H., Arnold E. (2009). Vitamin C supplementation for asthma. Cochrane Database Syst. Rev..

[105] Cohen H.A., Neuman I., Nahum H., Nahum M. (1997). Blocking Effect of Vitamin C in Exercise-Induced Asthma. Arch. Pediatr. Adolesc. Med..

[106] Nagel G., Linseisen J. (2005). Dietary Intake of Fatty Acids, Antioxidants and Selected Food Groups and Asthma in Adults. Eur. J. Clin. Nutr..

[107] Wang C.S., Wang J., Zhang X., Zhang L., Zhang H.P., Wang L., Yang C., Wang Y. (2018). Fast Food Consumption, Dietary Factors and Asthma Prevalence in Adolescents. Respirology.

[108] Al-Zalabani A.H., Elahi I.N., Katib A., Alamri A.G., Halawani A., Alsindi N.M., Alharbi K.K., Abduljabbar A.Z., Al-Dakheel F.M., Alzahrani A.S. (2019). Association between Soft Drinks Consumption and Asthma: A Systematic Review and Meta-Analysis. BMJ Open.

[109] Bisgaard H., Stokholm J., Chawes B.L., Vissing N.H., Bjarnadóttir E., Schoos A.M., Wolsk H.M., Pedersen T.M., Vinding R.K., Folsgaard N.V. (2016). Fish Oil-Derived Fatty Acids in Pregnancy and Wheeze/Asthma in Offspring. N. Engl. J. Med..

[110] Birch E.E., Khoury J.C., Berseth C.L., Castañeda Y.S., Couch J.M., Bean J., Tamer R., Harris C.L., Mitmesser S.H., Scalabrin D.M.F. (2010). The Impact of Early Nutrition on Incidence of Allergic Manifestations and Common Respiratory Illnesses in Children. J. Pediatr..

[111] Hodge L., Salome C.M., Peat J.K., Haby M.M., Xuan W., Woolcock A.J. (1996). Consumption of Oily Fish and Childhood Asthma Risk. Med. J. Aust..

[112] Antova T., Pattenden S., Nikiforov B., Leonardi G.S., Boeva B., Fletcher T., Rudnai P., Slachtova H., Tabak C., Zlotkowska R. (2003). Nutrition and Respiratory Health in Children in Six Central and Eastern European Countries. Thorax.

[113] Escamilla-Nuñez M.C., Barraza-Villarreal A., Hernández-Cadena L., Navarro-Olivos E., Sly P.D., Romieu I. (2014). Omega-3 Fatty Acid Supplementation during Pregnancy and Respiratory Symptoms in Children. Chest.

[114] Akter R., Afrose A., Sharmin S., Rezwan R., Rahman R., Neeletol S. (2022). A Comprehensive Look into the Association of Vitamin D Levels and Vitamin D Receptor Gene Polymorphism with Obesity in Children. Biomed. Pharmacother..

[115] Papamichael M.M., Itsiopoulos C., Katsardis C., Tsoukalas D., Erbas B. (2022). Does BMI Modify the Association between Vitamin D and Pulmonary Function in Children of the Mild Asthma Phenotype?. Int. J. Environ. Res. Public Health.

[116] Liu J., Dong Y.Q., Yin J., Yao J., Shen J., Sheng G.J., Li K., Lv H.F., Fang X., Wu W.F. (2019). Meta-Analysis of Vitamin D and Lung Function in Asthma Patients. Respir. Res..

[117] Majak P., Olszowiec-Chlebna M., Smejda K., Stelmach I. (2011). Vitamin D Supplementation in Children May Prevent Asthma Exacerbation Triggered by Acute Respiratory Infection. J. Allergy Clin. Immunol..

[118] Urashima M., Segawa T., Okazaki M., Kurihara M., Wada Y., Ida H. (2010). Randomized Trial of Vitamin D Supplementation to Prevent Seasonal Influenza A in Schoolchildren. Am. J. Clin. Nutr..

